# Changes in the presenting symptoms of lung cancer from 2000–2017: a serial cross-sectional study of observational records in UK primary care

**DOI:** 10.3399/bjgp20X708137

**Published:** 2020-01-28

**Authors:** Sarah Chowienczyk, Sarah Price, Willie Hamilton

**Affiliations:** College of Medicine and Health, University of Exeter, Exeter.; College of Medicine and Health, University of Exeter, Exeter.; College of Medicine and Health, University of Exeter, Exeter.

**Keywords:** cancer, cough, diagnosis, dyspnoea, lung cancer, observational study

## Abstract

**Background:**

Most patients diagnosed with lung cancer present with symptoms. It is not known if the proportions of patients presenting with each symptom has changed over time. Identifying trends in lung cancer’s presenting symptoms is important for medical education and early-diagnosis initiatives.

**Aim:**

To identify the first reported symptom of possible lung cancer (index symptom), and to test whether the percentages of patients with each index symptom changed during 2000–2017.

**Design and setting:**

This was a serial, cross-sectional, observational study using UK Clinical Practice Research Datalink (CPRD) data with cancer registry linkage.

**Method:**

The index symptom was identified for patients with an incident diagnosis of lung cancer in annual cohorts between 1 January 2000 and 31 December 2017. Searches were constrained to symptoms in National Institute for Health and Care Excellence (NICE) suspected-cancer referral guidelines, and to the year before diagnosis. Generalised linear models (with a binomial function) were used to test if the percentages of patients with each index symptom varied during 2000–2017.

**Results:**

The percentage of patients with an index symptom of cough (odds ratio [OR] 1.01; 95% confidence interval [CI] = 1.00 to 1.02 per year; *P*<0.0001) or dyspnoea (OR 1.05; CI = 1.05 to 1.06 per year; *P*<0.0001) increased. The percentages of patients with other index symptoms decreased, notably haemoptysis (OR 0.93; CI = 0.92 to 0.95; *P*<0.0001) and appetite loss (OR 0.94; CI = 0.90 to 0.97; *P*<0.0001).

**Conclusion:**

During 2000–2017, the proportions of lung cancer patients with an index symptom of cough or dyspnoea increased, while the proportion of those with the index symptom haemoptysis decreased. This trend has implications for medical education and symptom awareness campaigns.

## INTRODUCTION

In the UK, lung cancer is the leading cause of cancer deaths and has a 5-year age-standardised net survival rate of 13.3%.^1^^,^^2^ The prognosis of patients diagnosed with lung cancer is strongly related to stage, and survival is better for patients diagnosed through primary care than for those diagnosed through emergency routes.^3^^,^^4^ Therefore, much effort has been invested in improving the recognition of patients with suspected lung cancer presenting to primary care.

The symptoms of potential lung cancer reported by patients to their GPs and the positive predictive values of these symptoms have been identified.^5^ The National Institute for Health and Care Excellence (NICE) guidelines on the recognition and referral of suspected cancer were updated in 2015.^6^ NICE recommends an urgent referral for patients with haemoptysis or an urgent chest X-ray for patients with lower-risk symptoms.^6^

Current medical education still highlights haemoptysis as the cardinal symptom of lung cancer, and other symptoms such as dyspnoea and cough are downplayed.^7^ While patients are likely to recognise the significance of haemoptysis, awareness of other symptoms and of the significance of cough is relatively low.^8^ However, this may be changing, particularly among patients, not least because recent symptom awareness campaigns have encouraged the public to see their GP if they have a cough, and because of increased attribution of this symptom to lung cancer.^9^^–^11

The aims of this study of patients with lung cancer were to:

- identify patients with lung cancer symptoms recorded before their lung cancer diagnosis (any-time symptom);
- identify patients’ first recorded symptom (index symptom); and
- to test whether the percentages of patients with each index symptom changed from 2000–2017.

## METHOD

### Study design and population

This serial, cross-sectional, observational study used data from the Clinical Practice Research Datalink (CPRD) with linked data from the National Cancer Registration and Analysis Service (NCRAS). The CPRD is a large database of coded, anonymised, electronic medical records created during everyday consultations from >600 UK general practices.^12^ Patients were included if they:

- were aged ≥18 years;
- had an incident diagnosis of lung cancer made between 1 January 2000 and 31 December 2017; and
- were registered at a CPRD practice ≥1 year before diagnosis.

The CPRD excluded patients with a previous diagnosis of cancer recorded in their medical records using a comprehensive list of cancer codes. The research team excluded patients if they had multiple primary cancers diagnosed on the same day. The CPRD provided 27 889 potentially eligible participants; 94 patients with multiple primary cancers were excluded, leaving 27 795 eligible participants.

**Table:** How this fits in

The majority of patients with lung cancer present with symptoms; these symptoms and their positive predictive values for cancer have been identified. Using primary care records, this study looked for the first possible symptom of lung cancer (index symptom), and any trends over time in the proportions of patients with each index symptom. From 2000–2017, the percentages of patients with cough and dyspnoea as an index symptom increased. The percentages of patients with other index symptoms decreased, most notably for haemoptysis and appetite loss. Clinicians should be aware that, although haemoptysis remains an important symptom, its presentation is increasingly rare. Medical education needs to place at least as much emphasis on the more common symptoms of cough and dyspnoea as it does on haemoptysis.

### Cancer diagnosis

The cancer diagnosis date was derived from the earliest recorded cancer diagnostic code in the CPRD. Diagnosis type and date were validated by the linked NCRAS data where available, with the NCRAS date taking precedence where there were discrepancies. Only patients with an incident diagnosis of cancer were included.

### Symptoms

Lung cancer symptoms were those included in the NICE suspected-cancer referral guidelines, namely haemoptysis, cough, fatigue, dyspnoea, chest pain, weight loss, and appetite loss.^6^ Lists of Read Codes for these symptoms were collated using robust methods.^13^ Patients were classified as having experienced the symptom if they had a consultation with a Read Code corresponding to the symptom. ‘Any-time symptoms’ were defined as those that occurred in the year before diagnosis. The ‘index symptom’ was defined as the earliest recorded symptom. Multiple index symptoms on the same day were labelled as a ‘combination’ symptom, categorised by whether or not they included cough (the most prevalent symptom). In order to capture non-specified presentations, consultations coded as ‘suspected cancer’ or ‘abnormal chest X-ray’ were identified where they preceded any recorded named symptoms. For annual cohorts of patients from 2000–2017, the numbers (and percentages) of patients with each symptom (both index and any-time) were reported.

### Statistical analysis

The authors used a generalised linear model with a link function and a binomial probability distribution for the outcome variable, which was the number of patients with each index symptom. The explanatory variables were year of diagnosis (2000–2017), age, and sex. The model reported odds ratios (OR) for the outcome variable per year, with an OR>1.0 representing an increase over time, and an OR<1.0 representing a decrease over time.

## RESULTS

After exclusions, 27 795 patients (44.4% female) with lung cancer, with a mean age of 72 years (standard error of the mean = 0.06 years), were studied.

For each annual cohort, the numbers of patients with each index symptom and with each any-time symptom are shown in Tables 1 and 2, respectively. Of the 27 795 patients, 1233 (4.4%) had a non-specified primary care presentation of possible lung cancer occurring before a named symptom, of which 209 (0.8%) were an abnormal chest X-ray and 1024 (3.7%) were a suspected cancer code (Table 1). The percentage of patients with an abnormal chest X-ray preceding any symptoms decreased year on year (OR 0.89 per year; 95% confidence interval [CI] = 0.86 to 0.91; *P*<0.0001). In contrast, the percentage of patients with a suspected-cancer code preceding any symptoms increased year on year (OR 1.08; CI = 1.07 to 1.96; *P*<0.0001).

**Table 1.** Number of patients with each index symptom occurring in the year before their lung cancer diagnosis for each annual cohort

**Year of cancer diagnosis**	**Cohort**	**Cough, *N* (%)**	**Dyspnoea, *N* (%)**	**Chest pain, *N* (%)**	**Fatigue, *N* (%)**	**Haemoptysis, *N* (%)**	**Suspected cancer, *N* (%)**	**Weight loss, *N* (%)**	**Combination cough + other, *N* (%)**	**Abnormal chest X-ray, *N* (%)**	**Combination not including cough, *N* (%)**	**Appetite loss, *N* (%)**
**2000**	971	211 (22)	128 (13)	108 (11)	38 (4)	57 (6)	15 (2)	31 (3)	5 (1)	25 (3)	6 (1)	9 (1)
**2001**	1053	216 (21)	123 (12)	112 (11)	32 (3)	52 (5)	29 (3)	39 (4)	12 (1)	16 (2)	9 (1)	10 (1)
**2002**	1174	251 (21)	163 (14)	132 (11)	42 (4)	73 (6)	23 (2)	36 (3)	5 (0)	22 (2)	7 (1)	11 (1)
**2003**	1297	288 (22)	152 (12)	121 (9)	73 (6)	81 (6)	25 (2)	38 (3)	14 (1)	16 (1)	7 (1)	9 (1)
**2004**	1624	367 (23)	200 (12)	154 (9)	74 (5)	84 (5)	43 (3)	47 (3)	28 (2)	12 (1)	17 (1)	11 (1)
**2005**	1712	420 (25)	211 (12)	153 (9)	72 (4)	80 (5)	32 (2)	49 (3)	30 (2)	10 (1)	15 (1)	11 (1)
**2006**	1809	443 (24)	218 (12)	186 (10)	58 (3)	76 (4)	55 (3)	60 (3)	42 (2)	18 (1)	23 (1)	17 (1)
**2007**	1900	489 (26)	258 (14)	158 (8)	87 (5)	68 (4)	65 (3)	72 (4)	32 (2)	11 (1)	19 (1)	13 (1)
**2008**	1951	523 (27)	257 (13)	173 (9)	74 (4)	72 (4)	54 (3)	69 (4)	46 (2)	13 (1)	10 (1)	16 (1)
**2009**	1874	490 (26)	290 (15)	146 (8)	84 (4)	61 (3)	74 (4)	77 (4)	43 (2)	19 (1)	7 (0)	9 (0)
**2010**	1837	447 (24)	388 (21)	135 (7)	67 (4)	52 (3)	66 (4)	41 (2)	50 (3)	7 (0)	13 (1)	14 (1)
**2011**	1925	485 (25)	426 (22)	149 (8)	55 (3)	76 (4)	88 (5)	48 (2)	41 (2)	4 (0)	11 (1)	11 (1)
**2012**	1840	483 (26)	331 (18)	143 (8)	67 (4)	54 (3)	85 (5)	47 (3)	48 (3)	9 (0)	14 (1)	9 (0)
**2013**	1830	446 (24)	355 (19)	111 (6)	74 (4)	39 (2)	93 (5)	57 (3)	72 (4)	3 (0)	12 (1)	12 (1)
**2014**	1586	404 (25)	305 (19)	97 (6)	49 (3)	42 (3)	79 (5)	40 (3)	48 (3)	11 (1)	14 (1)	3 (0)
**2015**	1417	338 (24)	287 (20)	90 (6)	49 (3)	35 (2)	82 (6)	31 (2)	66 (5)	5 (0)	8 (1)	2 (0)
**2016**	1095	273 (25)	228 (21)	58 (5)	47 (4)	25 (2)	53 (5)	30 (3)	27 (2)	5 (0)	6 (1)	5 (0)
**2017**	900	207 (23)	197 (22)	57 (6)	22 (2)	16 (2)	63 (7)	17 (2)	22 (2)	3 (0)	7 (1)	2 (0)
**Total**	**27 795**	**6781**	**4517**	**2283**	**1064**	**1043**	**1024**	**829**	**631**	**209**	**205**	**174**

**Table 2.** Number of patients who reported each symptom or any symptom in the year before their lung cancer diagnosis for each annual cohort

**Year of diagnosis**	**Cohort, *N* (%)**	**Any symptom, *N* (%)**	**Cough, *N* (%)**	**Dyspnoea, *N* (%)**	**Chest pain, *N* (%)**	**Haemoptysis, *N* (%)**	**Weight loss, *N* (%)**	**Fatigue, *N* (%)**	**Appetite loss, *N* (%)**
**2000**	971	601 (62)	263 (27)	195 (20)	161 (17)	109 (11)	57 (6)	69 (7)	21 (2)
**2001**	1053	614 (58)	288 (27)	201 (19)	174 (17)	104 (10)	67 (6)	56 (5)	25 (2)
**2002**	1174	724 (62)	317 (27)	255 (22)	196 (17)	121 (10)	59 (5)	71 (6)	23 (2)
**2003**	1297	788 (61)	379 (29)	278 (21)	192 (15)	133 (10)	71 (5)	101 (8)	36 (3)
**2004**	1624	993 (61)	484 (30)	352 (22)	241 (15)	164 (10)	101 (6)	119 (7)	34 (2)
**2005**	1712	1045 (61)	555 (32)	382 (22)	247 (14)	166 (10)	98 (6)	134 (8)	30 (2)
**2006**	1809	1131 (63)	602 (33)	406 (22)	305 (17)	157 (9)	120 (7)	109 (6)	44 (2)
**2007**	1900	1204 (63)	646 (34)	442 (23)	274 (14)	160 (8)	122 (6)	144 (8)	36 (2)
**2008**	1951	1244 (64)	680 (35)	462 (24)	269 (14)	142 (7)	117 (6)	128 (7)	36 (2)
**2009**	1874	1215 (65)	653 (35)	515 (27)	247 (13)	119 (6)	138 (7)	149 (8)	34 (2)
**2010**	1837	1212 (66)	643 (35)	630 (34)	253 (14)	135 (7)	91 (5)	129 (7)	39 (2)
**2011**	1925	1310 (68)	697 (36)	671 (35)	249 (13)	157 (8)	101 (5)	107 (6)	37 (2)
**2012**	1840	1211 (66)	670 (36)	563 (31)	250 (14)	128 (7)	90 (5)	107 (6)	27 (1)
**2013**	1830	1188 (65)	660 (36)	599 (33)	221 (12)	107 (6)	113 (6)	119 (7)	34 (2)
**2014**	1586	1009 (64)	583 (37)	484 (31)	188 (12)	80 (5)	81 (5)	83 (5)	25 (2)
**2015**	1417	914 (65)	519 (37)	460 (32)	167 (12)	77 (5)	62 (4)	85 (6)	15 (1)
**2016**	1095	706 (64)	388 (35)	343 (31)	112 (10)	60 (5)	53 (5)	68 (6)	14 (1)
**2017**	900	552 (61)	293 (33)	301 (33)	100 (11)	39 (4)	38 (4)	41 (5)	4 (0)
**Total**	**27 795**	**17 661**	**9320**	**7539**	**3846**	**2158**	**1579**	**1819**	**514**

Of the 27 795 patients, 17 661 (63.5%) had a recorded lung cancer symptom. Cough was the most common index (*n* = 6781, 24.4%) or any-time (*n* = 9320, 33.5%) symptom.

**Figure 1.**
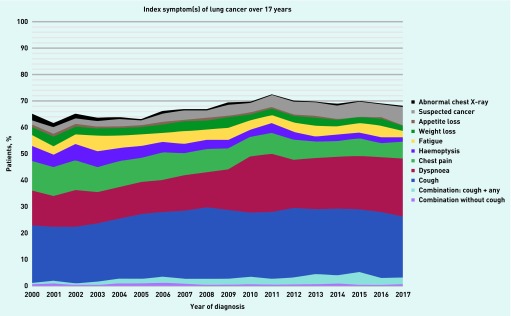
***Index symptoms of lung cancer, 2000–2017.***

**Figure 2.**
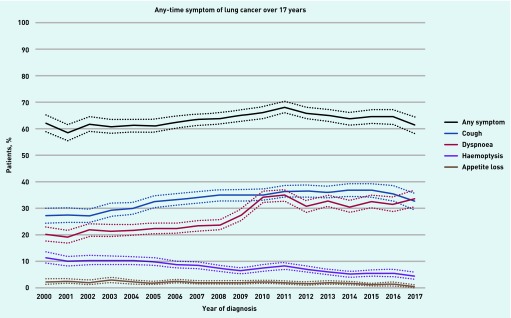
***Trends in any occurrence of cough, shortness of breath, appetite loss, or haemoptysis in the year before diagnosis, by annual cohort.^a^*** ***^a^Trends in the percentage of patients with any symptoms of possible lung cancer are also shown for reference. Values are percentages, and 95% confidence intervals are indicated by the dotted lines.***

The percentages of patients with index symptoms of dyspnoea or cough (alone or in combination with another symptom) increased over time (Figure 1). Conversely, the other index symptoms, most notably appetite loss and haemoptysis, became less common over time (Figure 1). The percentages of patients with dyspnoea and cough as any-time symptoms increased over time and the percentages of patients with haemoptysis and appetite loss as any-time symptoms decreased over time (Figure 2).

The analysis showed a statistically significant year-on-year increase in the percentages of patients with index symptoms of dyspnoea (OR 1.05; CI = 1.05 to 1.06; *P*<0.0001) or cough (OR [cough alone] 1.01; CI = 1.00 to 1.02, *P*<0.0001; OR [cough plus another index symptom] 1.08; CI = 1.06 to 1.10; *P*<0.0001) (Table 3). There was a statistically significant decrease year-on-year in the percentages of patients with index symptoms of haemoptysis (OR 0.93; CI = 0.92 to 0.95, *P*<0.0001), appetite loss (OR 0.94; CI = 0.90 to 0.97, *P*<0.0001), chest pain (0.96; CI = 0.95 to 0.97; *P*<0.0001), or weight loss (OR 0.98; CI = 0.96 to 0.99; *P* = 0.004) (Table 3).

**Table 3.** Number of patients with each index symptom in the year before diagnosis with lung cancer^a^

**Index symptom**		**Number of patients diagnosed in 2000, *N*= 971 (%)**	**Number of patients diagnosed in 2017, *N*= 900 (%)**	**OR per year (95% CI)**	***P*-value**
**Increasing over time**	Combination (more than one symptom including cough)	5 (1)	22 (2)	1.08 (1.06 to 1.10)	<0.0001
	Dyspnoea	128 (13)	197 (22)	1.05 (1.05 to 1.06)	<0.0001
	Cough	211 (22)	207 (23)	1.01 (1.00 to 1.02)	<0.0001

**Decreasing over time**	Fatigue	38 (4)	22 (2)	0.99 (0.97 to 1.00)	0.0270
	Combination (more than one symptom excluding cough)	6 (1)	7 (1)	0.98 (0.96 to 1.01)	0.3040
	Weight loss	31 (3)	17 (2)	0.98 (0.96 to 0.99)	0.004
	Chest pain	108 (11)	57 (6)	0.96 (0.95 to 0.97)	<0.0001
	Appetite loss	9 (1)	2 (0)	0.94 (0.90 to 0.97)	<0.0001
	Haemoptysis	57 (6)	16 (2)	0.93 (0.92 to 0.95)	<0.0001

**Total**		**593 (61)**	**547 (61)**		

a For brevity, data for the intervening annual cohorts 2001–2016 are not shown. The odds ratio reports the change in proportion of patients with each index symptom per year, from 2000–2017. OR = odds ratio.

## DISCUSSION

### Summary

The percentages of patients presenting with symptoms of dyspnoea or cough before their lung cancer diagnosis increased from 2000–2017. Conversely, the percentages of patients with lung cancer presenting with symptoms of haemoptysis or appetite loss declined, such that haemoptysis and appetite loss are now very rare presenting symptoms of lung cancer.

### Strengths and limitations

The strengths of this study include the NCRAS linkage and the robust methods used for collating comprehensive code lists for symptoms.^13^ This study is limited by its use of Read Codes to establish if a patient has experienced a symptom. The Read Codes do not provide information about the duration and severity of symptoms or whether it was patient reported or doctor elicited. Some patients will have been misclassified if symptoms were not recorded or were noted in an irretrievable part of the medical records. Symptoms described in the free text were inaccessible. There is evidence that this may bias estimates in favour of established red-flag symptoms, such as haemoptysis, which are more likely than ‘low-risk but not no-risk’ symptoms to be recorded using codes.^14^ Furthermore, a small number of patients had a non-specified presentation to primary care with ‘suspected cancer’ or abnormal chest X-ray codes, and these could not be classified further.

Changes in coding practices and in the profile of general practices contributing data to the CPRD over time could have altered the estimates of the prevalence of recorded symptoms. Reassuringly, the proportion of patients with any recorded symptom was relatively constant over time, suggesting that any such changes were minor. The symptoms of lung cancer are shared with comorbidities, in particular chronic obstructive pulmonary disease (COPD). In theory, changes in the primary care management of COPD could have led to differences in symptom recording. The main driver of change in primary care COPD management was the Quality and Outcomes Framework introduced in 2004, which encouraged documentation of chronic disease.^15^ However, there is no suggestion from Figure 1 that symptoms changed markedly around this date.

### Comparison with existing literature

To the authors’ knowledge this is the first study to examine changes in the presenting symptoms of lung cancer over time. Previous studies have also concluded that the commonest symptoms of lung cancer are cough and dyspnoea.^16^^–^19 In support of this study’s findings, Iyen-Omofoman *et al*^17^ found that only 248 of 12 074 (2.1%) patients with lung cancer diagnosed from 2000–2009 experienced haemoptysis. Most other studies conclude that haemoptysis is more frequent, however, occurring in between 8.8% and 22.0% of patients before diagnosis.^16^^,^^18^^,^^19^ However, some of those studies were set in secondary care (where the symptom profiles differ markedly from those in primary care); were from a much earlier period (1998–2002); had smaller sample sizes; or examined longer pre-diagnostic intervals.^16^^,^^18^^,^^19^

### Implications for research and practice

Future research is needed to investigate the association between symptoms and patient outcomes in order to explore if the observed trend in symptomatology represents earlier presentation of lung cancer. Continued research of patient factors influencing help-seeking behaviour for specific lung cancer symptoms would help improve understanding of the observed trends in symptomology. Specifically, it would be valuable to know if patients are becoming increasingly knowledgeable about lung cancer symptoms and whether this means that they are more willing to consult their GP about their symptoms.

Haemoptysis remains an important presenting symptom of lung cancer as it has the highest positive predictive value.^5^ It is often thought of as the main presenting symptom of lung cancer, with a recent review stating that haemoptysis occurs in 20% of patients with lung cancer.^8^ This study suggests that haemoptysis is actually a very rare symptom, and is becoming even more uncommon. Therefore, prominence should be given to other lung cancer symptoms in undergraduate and postgraduate education. Given that cough and dyspnoea are the most common presenting symptoms, continued targeting of these symptoms in public awareness campaigns may help to improve lung cancer diagnosis.

In summary, cough and dyspnoea are the most common presenting symptoms of lung cancer and from 2000–2017 the proportion of patients presenting with these symptoms has increased. In contrast, haemoptysis and appetite loss are now relatively rare presenting symptoms of lung cancer. Symptom awareness campaigns should target cough and dyspnoea. In addition, medical education needs to change, to avoid clinicians overlooking the importance of cough and dyspnoea as symptoms of undiagnosed lung cancer.
